# Evaluation of Indigenous *Candida oleophila* and *Candida boidinii* in Monoculture and Sequential Fermentations: Impact on Ethanol Reduction and Chemical Profile in Chilean Sauvignon Blanc Wines

**DOI:** 10.3390/jof8030259

**Published:** 2022-03-03

**Authors:** Sergio Benavides, Wendy Franco, Consuelo Ceppi De Lecco, Angélica Durán, Alejandra Urtubia

**Affiliations:** 1 Núcleo de Investigación en Agroalimentos y Nutrición Aplicada, Universidad Adventista de Chile, Camino a las Mariposas km 12, Chillán 3780000, Chile; sergiobenavides@unach.cl; 2 Departamento de Ingeniería Química y Bioprocesos, Pontificia Universidad Católica de Chile, Ave. Vicuña Mackena 4860, Santiago 7820244, Chile; wfranco@ing.puc.cl; 3 Departamento de Ciencias de la Salud, Carrera de Nutrición y Dietética, Pontificia Universidad Católica de Chile, Ave. Vicuña Mackena 4860, Santiago 7820244, Chile; 4 Departamento de Fruticultura y Enología, Pontificia Universidad Católica de Chile, Ave. Vicuña Mackena 4860, Santiago 7820244, Chile; ceppidelecco@uc.cl; 5 Departamento de Ingeniería Química y Ambiental, Universidad Técnica Federico Santa María, Ave. España 1680, Valparaíso 2390123, Chile; angelica.duran@usm.cl

**Keywords:** non-*Saccharomyces*, *Candida* sp., wine fermentation, ethanol reduction

## Abstract

The study of non-*Saccharomyces* yeasts in wine fermentations allows the exploration of new alternatives for the reduction of ethanol in wines. The objective of this work was to evaluate the fermentation capacity of two indigenous *Candida* yeasts (*C. oleophila* and *C. boidinii*) in monoculture and sequential fermentations (laboratory and microvinification scale) to produce Chilean Sauvignon Blanc wine. Fermentations were monitored by the determination of ethanol, glycerol, organic acids, and residual sugars. The results indicated that at the laboratory scale for both the monoculture and sequential fermentations it was possible to reduce the ethanol concentration on 0.77% *v*/*v* (monoculture) and 1.5% *v*/*v* (sequential) for *C. oleophila* and 0.50% *v*/*v* (monoculture) and 0.04% *v*/*v* (sequential) for *C. boidinii* compared to *S. cerevisiae* (12.87% *v*/*v*). Higher glycerol concentrations were produced in monoculture than sequential fermentations (*C. oleophila*: 9.47 g/L and *C. boidinii* 10.97 g/L). For microvinifications, the monoculture and sequential fermentations with *C. boidinii* managed to reduce ethanol content by 0.17% *v*/*v* and 0.54% *v*/*v*, respectively, over the *S. cerevisiae* control (13.74% *v*/*v*). In the case of *C. oleophila*, the reduction was only observed in sequential fermentations with 0.62% *v*/*v*. Interestingly, grapes with higher sugar concentration resulted in wines with lees ethanol concentrations. This might be associated to the use of *C. oleophila* (13.12% *v*/*v*) and *C. boidinii* (13.20% *v*/*v*) in sequential fermentations microvinification scale.

## 1. Introduction

In recent years, there has been a significant increase in the demand for more exclusive wines, with innovative sensory profiles, and even lower ethanol content [1]. This has forced the wine industry to pursue winemaking alternatives, which include the agricultural management of vineyards, fermentation conditions, and more recently, the exploration of yeasts other than *Saccharomyces cerevisiae*, which certainly poses a significant challenge within the area [2,3]. While it is true that there is a significant number of articles that explore the use of non-*Saccharomyces* yeasts (NSY), it is also true that the microbiology of grapes is complex and dependent on climate and regional factors [4,5]. This means that there is a large number of species and subspecies of yeast that have not been isolated neither characterized, each with fermentation properties that might positively influence in the wine attributes.

Most scientific articles associated with the study of NSY indicate that these yeasts have a positive impact on the sensory quality of wine [2], and in some cases on the reduction in ethanol content [6]. NSY have an interesting winemaking profile due to their capacity to generate compounds of high sensory interest, such as glycerol, acetaldehyde, terpenes, esters, or organic acids, whose combination generates an aromatic profile of high complexity and unique characteristics [1,7]. Up to now, over one-hundred different species of NSY have been identified, although only a fraction of these have been evaluated in terms of their fermentation capacity. Moreover, studies have focused in characterizing the yeasts using synthetic and/or natural grape juice at laboratory scale, however little is known at lager scales that mimic the winemaking conditions used at a vineyard [8].

The most common NSY studied are *Metschnikowia pulcherrima*, *Lachancea thermotolerans*, *Torulaspora delbrueckii*, and *Pichia kluyveri* [7,8], which have been industrialized and are sold in the form of commercial starter cultures. Nevertheless, there are other genera and species of potential interest that have not been greatly explored. An interesting genus of NSY within this group are the *Candida* sp. These yeasts have been reported as active participants in spontaneous fermentations, showing resistance to ethanol [9]. However, with the exception of species *C. zemplinina, C. californica*, or *C. famata* [10,11,12], this group has been scarcely studied and reported. *Candida* yeasts associated with wine fermentations are characterized as being a good producer of glycerol and poor producer of acetic acid compared to traditional starter [9,13,14].

On the other hand, the yeasts can produce enzymes that favor the formation of desirable aromatic compounds [14]. *C. famata* can produce high concentration of D-glucosidase, which improved the aromatic profile of muscatel wines by the release of monoterpenoids [15]. Moreover, *C. famata* can produce exopolysaccharides that positively influences the texture and astringency of wine [14]. On the other hand, the *C. californica* has been reported as tolerant to ethanol, and with an interesting hydrolase activity, properties that in sequential fermentations with *S. cerevisiae* resulted in wines with good aromatic profiles [13,16]. However, there are other *Candida* species that, to our knowledge, have been little studied for wine fermentations, and thus their fermentation potential is still unknown, such is the case of *C. oleophila* and *C. boidinii*. For this reason, and also considering the attractive fermentation potential of the *Candida* yeasts, the objective of this work was to evaluate the fermentation potential of these NSY for the production of Sauvignon Blanc wine made with grapes grown in Chile. For this, the yeasts fermentation capacity was evaluated at laboratory-scale (500 mL) and microvinifications (10 L) following two approaches: monoculture and sequential fermentations. The impact on the chemical fermentation profile and ethanol content was evaluated.

## 2. Materials and Methods

### 2.1. Yeasts

The *C. oleophila* (LVNS 2) and *C. boidinii* (LVNS 9) yeasts were acquired from the culture collection at the Food Fermentation Laboratory (Pontificia Universidad Católica de Chile/Universidad Técnica Federico Santa María). These yeasts were isolated from grapes collected from vineyards in the Maule Region of Chile (35°26′ S 71°40′ W) and identified through partial sequencing of the 26S *rDNA* [9]. For the laboratory experiments, the yeast cultures were kept in agar plates, while for the microvinification the yeasts were lyophilized. This was done in order to emulate the conditions at which yeast are commonly used in the vineyards.

For the lyophilization, colonies of each yeast were grown in Sabouraud broth (SB, Biokar, Barcelona, España) at 28 °C for 24 h in an incubator (Pol-Eko SMAR, CL series, Wodzisław, Polony). After this time, the mix was centrifuged (Centurion 1.k1015 110 V 60 hz, Chichester, UK) at 4000 rpm for 5 min, and the supernatant was discarded. The biomass pellet was washed with saline solution (0.9% p/v). The clean pellet was then frozen (Haier, 92/262, Okara, Pakistán) at −40 °C for 48 h. The frozen mix was covered with a perforated aluminum sheet to facilitate water sublimation, and placed in a freeze dry (Liobras, L108, Sao Paulo, Brazil) at −50 °C with a pressure of 100 ± 25 μm Hg for 24 h. The freeze-dried yeast was stored at room temperature, protected from light exposure.

### 2.2. Evaluation of the Fermentation Potential in Glucose/Fructose Medium

#### 2.2.1. Glucose/Fructose Medium

The first step for evaluating the fermentation potential of the yeasts, was to determine the capacity to use monosaccharides in a glucose/fructose (G/F) synthetic medium. The medium was composed of yeast extract (7 g/L), glucose (75 g/L), and fructose (75 g/L), and the pH was adjusted to 3.5 [17,18].

#### 2.2.2. Starter Culture

Yeast colonies were incubated in 40 mL of SB broth (Biokar, Pantin, France), at 28 °C for 24 h. Subsequently, the culture was centrifuged at 6000 rpm for 15 min. The supernatant was discarded, and the pellet was washed twice with peptone water (1%). The pellet was re-suspended with the glucose/fructose medium. Yeast concentration was adjusted to approximately10^6^ UFC/mL.

#### 2.2.3. Fermentation

One-hundred milliliters of the G/F medium was inoculated (1% *v*/*v*) with the active yeasts culture (10^6^ UFC/mL). The mix was incubated at 25 °C, 200 rpm, for 5 days. As a control, a *S. cerevisiae* commercial yeast was used (Actiflore, Laboratorio Laffort, E1118, Bourdeux, France) at a dose of 20 g/hL (manufacturer’s recommendation). Fermentation process was monitored by the determination of cell concentration, sugar consumption and ethanol production. For cell enumeration, sample´s dilutions were measured using an automated cell counter (Bio-Rad TC20™, Hercules, CA, USA) following the manufacturer’s instructions. The ethanol, glycerol, and lactic acid were determined by high-pressure liquid chromatography (HPLC) (Agilent Technologies, Infinity 1260, Waldbronn, Germany), using a 300 × 7.8 mm Aminex HPX-87 chromatography column (BIORAD, Hercules, CA, USA), operating with a 210 nm wavelength DAD detector. The measurements were taken with a run time of 30 min and a sample injection volume of 20 μL; the mobile phase of H_2_SO_4_ with a concentration of 0.005 mMol and a flow of 0.6 mL/min. The glucose, fructose, malic acid and acetic acid were determined by enzymatic kits (Megazyme, Wicklow, Ireland). The compounds are identified via series of enzymatic reactions, measured by spectrophotometry at 340 nm, and 0.1 mL of sample volume.

### 2.3. Laboratory-Scale Fermentations

#### 2.3.1. Grape Juice

Sauvignon Blanc grapes harvested in the Casablanca Valley, Valparaiso Region, Chile (33° 19′ S 71°25′ W), were used for the grape juice preparation. The grapes were cleaned of plant debris and leaves, and pressed to obtain the juice, which was then filtered with a fine gauze to eliminate large particulates. The grape juice obtained had the following characteristics: density 1.088 g/mL; 22,8°Brix; Glucose 143 g/L; Fructose 136 g/L. The nitrogen content was adjusted to 250 mg/L of YAN with Diammonium Phosphate (DAP) salts (Sigma Aldrich, Saint Louis, MO, USA). Additionally, 30 mg/L of sodium metabisulfite were added to reach 30 ppm of free sulfites, measured by SO_2_ titration (HI 84,500 Hanna Instruments, Woonsocket, RI, USA). The conditioned grape juice was decanted in a refrigerator at 5 °C for 16 h. Upon decanting, the grape juice was characterized in terms of °Brix (Refractometer HI 96,801 Hanna Instruments, Woonsoket, RI, USA), density, and probable ethanol content.

#### 2.3.2. Fermentation

Two fermentation strategies were performed: monoculture (*C. oleophila* and *C. boidinii* separately) and sequential fermentations. For sequential fermentation, *Candida* yeasts were inoculated at the beginning of the fermentation, and once ~50% of sugars were consumed a commercial *S. cerevisiae* (Actiflore) yeast was added.

#### 2.3.3. Monoculture Fermentation

The conditioned grape juice was portioned in 500 mL two-mouth Erlenmeyer flasks provided with airlocks and inoculated with the active yeasts as described in Section 2.2.2. As a control, a commercial *S. cerevisiae* (Actiflore) yeast was used which was activated and inoculated according to the manufacturer’s instructions.

Following inoculation, the flasks were aerated for 2 min, and then placed in a shaker (Mod JSSI-100C, JS Research, Chungcheongnam, Korea) at 15 °C and 200 rpm for up to 15 days.

#### 2.3.4. Sequential Fermentation

For sequential fermentation, the same process described in Section 2.3.2. was followed, with the difference that when 50% of the fermentable sugars had been consumed, the *S. cerevisiae* (Actiflore) was inoculated as indicated in Section 2.3.2.

#### 2.3.5. Fermentation Follow-Up and Control

For both the monoculture and sequential fermentations, density, °Brix, and cell concentration were determined. The ethanol, glucose, fructose, glycerol, and organic acids concentrations of the fermentations were measured as described in point Section 2.2.3. The fermentations were assumed to be finalized when the density was less than 0.990 g/mL, or constant for up to 48 h.

### 2.4. Microvinifications

For execution of the microvinifications, the Sauvignon Blanc grapes were acquired from 2 geographic regions of Chile: Casablanca Valley, Valparaiso Region (33°19′ S 71°25′ W) and Curicó Valley, Maule Region (35°26′ S 71°40′ W). The bunches were submitted to a visual sanitary evaluation to discard the presence of *Botrytis* sp. The grapes were selected and kept refrigerated until the moment of grape juice extraction.

The day of experimentation the stems were removed. The clean grapes were then cold-pressed, and the grape juice obtained was subject to sulfitation with sodium metabisulfite (Sigma-Aldrich, Milwaukee, WI, United States of America), to reach 10 to 20 ppm of free sulfite. Next, the grape juice was refrigerated c for approximately 24 h, to avoid any plant debris in suspension, until a turbidity of 120–150 NTU (Nephelometric Turbidity Unit, Santiago, Chile) was obtained. Three fermentation units were used for each treatment, each one with a volume on 10 L.

The inoculums were prepared by mixing 0.5 g of each freeze-dried yeast in 40 mL of fresh Sauvignon Blanc grape juice and kept at 25 °C for 20 min. The active yeasts were inoculated in each flask at a concentration of 10^6^ cells/mL. Each yeast was evaluated separately. As a control, a commercial *S. cerevisiae* (Actiflore) yeast was used, which was activated and inoculated according to the manufacturer’s instructions.

The fermentation was monitored until the fermented grape juice reached a residual sugar concentration of 2 g/L or less, a density of approximately 0.990 g/mL with no variation for 48 h. During the follow-up, the production of ethanol and glycerol was evaluated, as well as the concentration of organic acids (malic and acetic), glucose, and fructose, following the procedures established in point Section 2.2.3.

### 2.5. Statistical Analysis

All fermentations were performed in triplicate, using a completely randomized experimental design. The data sets were analyzed through analysis of variance (ANOVA), and the means differences were determined with the Least Square Differences (LSD). The statistical software Statgraphics Centurion XIX version: 15.2.05 (Madrid, Spain) was used.

## 3. Results and Discussions

The results achieved from this work allowed the evaluation of the fermentation potential of *C. oleophila* and *C. boidinii*. In this regard, the effects on fermentation were established at two different scales (laboratory, 500 mL; and microvinification, 10 L) with two types of fermentation systems (monoculture and sequential).

### 3.1. Fermentation in the Synthetic (Glucose/Fructose) Medium

With the aim to determine the capacity of the yeasts to consume sugars and produce ethanol, the fermentation capacity of the *Candida* sp. in a synthetic glucose–fructose medium was evaluated.

Table 1 shows the fermentation results for each yeast based on fermentation yield and final ethanol production. The results indicated a significant difference in terms of both ethanol production and yield. *C. oleophila* showed the lowest yield (0.25 g ethanol/g glucose), with a final production of ethanol averaged at 4.20% *v*/*v*. For *C. boidinii*, the concentration of ethanol reached 6.4% *v*/*v*, significantly higher than *C. oleophila*. Interestingly, *C. oleophila* managed only to consume over 71% of the available sugars, while the *C. boidinii* consumed over 99%, which correlates with higher ethanol production. However, both had similar yields (0.25 and 0.24 g ethanol/g sugar, respectively). As expected, all of the yeasts showed much lower yields than the control yeast (*S. cerevisiae*). NSY have the capacity to generate divergent routes of carbon flow, increasing the production of byproducts and/or biomass as opposed to the production of ethanol. Additionally, anaerobic or limited oxygen conditions further increase the production of secondary compounds leading to lower ethanol production with respect to traditional yeasts [19].

Sugar consumption is different for *C. oleophila* (71.77%) and *C. boidinii* (99.89%), and it can be explained by the differences in metabolism that are manifested in each strain. Contreras et al. (2014) reported that even yeasts belonging to the same genera showed different ethanol yields [17]. Some studies have indicated that NSY have metabolic preferences for glucose or fructose, which generates differences between the fermentation rates of both types of sugar. This metabolic preference can be attributed to phenomena of differential transport through the plasma membrane, form of phosphorylation of hexoses at the intracellular level, and other metabolic phenomena [20]. Additionally, all the yeast under study showed different ethanol yields to those reported in the literature. Nonetheless, the metabolic performance of yeasts, as well as microorganisms, are conditioned by several factors that can affect the metabolism, such as nutrients, aeration, and temperature. When a medium only with glucose/fructose is used, with very low presence of other nutrients (especially vitamins and nitrogen sources), the metabolic rate can be affected [21,22].

### 3.2. Fermentations at Laboratory Scale

#### 3.2.1. Monoculture Fermentation

**Figure 1:**
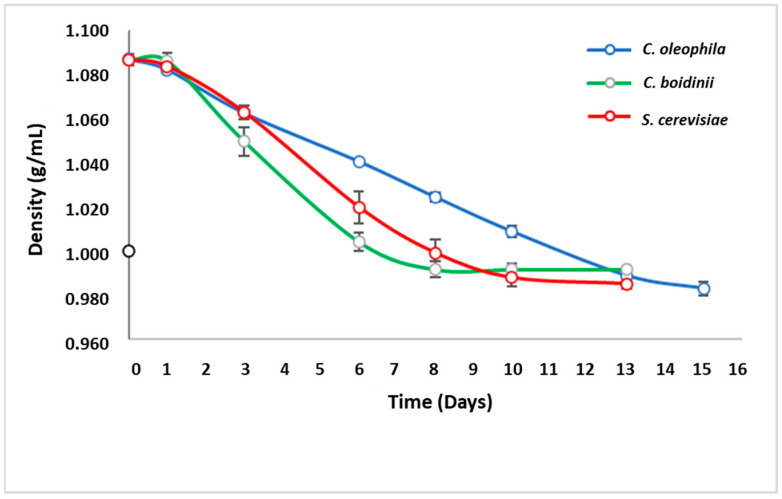
Evolution of substrate consumption through the measurement of density in monoculture fermentations (Sauvignon Blanc).

**Figure 2:**
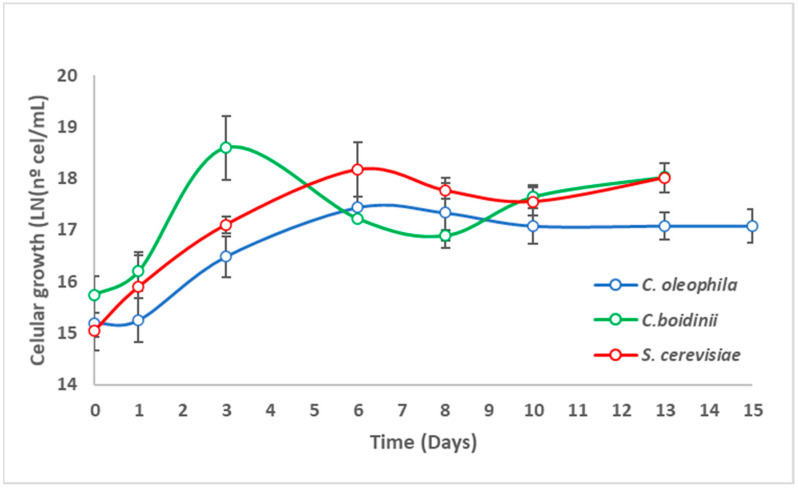
Evolution of cell proliferation in monoculture fermentations (Sauvignon Blanc).

With the purpose to evaluate the fermentation capacity of the *Candida* yeasts in real grape juice, monoculture fermentations were then performed at the laboratory level. The Sauvignon Blanc grape juice used was acquired from Casablanca Valley (Valparaíso, Chile). The juice had a total of soluble solids of 22.8 °Brix. Monoculture fermentation process was evaluated by monitoring of density (Figure 1), cell growth (Figure 2), and the final composition of the grape juice upon fermentation (Table 2 and Table 3).

As it was expected, *S. cerevisiae* (control) had a high capacity for sugar consumption, which correlated with the rapid density drop (Figure 1). Similarly, *C. boidinii* was just as effective in sugar consumption as the control. However, the control sample finished the fermentation process in 10 days, while *C. boidinii* in 13 days, reaching final density values of around 0.992 g/mL. Meanwhile, the fermentation with *C. oleophila* took 15 days to be completed, showing a constant and slower rate of sugar consumption (Figure 1).

In parallel, cell growth (Figure 2) was consistent with density. All the yeasts showed very brief or non-existent lag phase. In fact, the exponential growth began between day zero (*S. cerevisiae*) and day 1 (*C. boidinii* and *C. oleophila*). Nevertheless, the duration of the exponential phase varied significantly in some cases. In the control (*S. cerevisiae*), the exponential growth went from approximately day 0 to 6 days, which was similar to the case of *C. oleophila*, whose exponential phase went from day 1 to 6. In the case of the *C. boidinii* this period was much shorter, lasting only from day 1 to day 3 (Figure 2).

Regarding to the products and metabolites generated and consumed at the end of the fermentations, they are shown in Table 2 and Table 3. In terms of ethanol production, there were significant differences within the NSY studied, as well as with the control (*S. cerevisiae*). As expected, the higher ethanol production was observed for the control, whose final concentration was 12.87% *v*/*v*. On the other hand, *C. boidinii* produced 12.37% *v*/*v* and *C. oleophila* generated 12.10% *v*/*v*. These results suggest that the use of the two *Candida* are a suitable starter for the of wines with a reduced ethanol content using *Candida* yeasts, which is also consistent with several studies on the use of NSY. Aplin et al. (2019) obtained a reduction of 0.8% *v*/*v* when *C. californica* was used to ferment grape juice [10]. Similarly, Contreras et al. (2015) reported ethanol reduction for several NSY, being of particularly interesting *M. pulcherrima*, which reduced 0.9 and 1.6% *v*/*v* in Chardonnay and Shiraz grapes juices, respectively [6]. In the same way, Hranilovic et al. (2020) reported a reduction of 1.6% *v*/*v* in Shiraz in both monoculture and sequential fermentations for several strains of *M. pulcherrima* [23].

It is necessary to note the differences in ethanol production between the glucose/fructose medium and the Sauvignon Blanc grape juice. In this sense, the control (*S. cerevisiae*) in the glucose–fructose medium (Table 1) produced 13.90% *v*/*v* of ethanol, while *C. oleophila* (4.20% *v*/*v*) and *C. boidinii* (6.40% *v*/*v*) produced significantly lower concentrations. However, the difference in ethanol production in the Sauvignon Blanc grape juice (Casablanca Valley) did not shown great differences (0.77 and 0.50% *v*/*v* less ethanol than the control for *C. oleophila* and *C. boidinii*, respectively). This suggests that the nutritional complexity of the grape juice has significant influence over the metabolic rates of the *Candida* yeasts. In this case, note that the glucose/fructose medium contains very low amounts of assimilable nitrogen, which is a key nutritional factor that influences not only cell growth, but also fermentation kinetics. A low availability of assimilable nitrogen negatively affects protein synthesis, sugar transport and the biosynthesis of alcohols and esters. In relation to the efficiency in sugar use during the fermentation, it was observed that all yeasts tested consumed a high amount of sugar. This could be observed in the low residual sugar at the end of the fermentation, which was around 2.17 g/L (*C. boidinii*) and 2.70 g/L (*C. oleophila*), showing no significant differences with the control (*S. cerevisiae*) whose residual sugar content was 2.50 g/L. These results indicate that the inclusion of the yeasts studied results in the consumption of the grape juice sugars. However, as the must is not sterile, we assume that the presence of other yeasts contribute to the consumption of sugars in the must.

Table 3 shows the results of secondary compounds of fermentation. The production of acetic acid was consistent with those found by García-Fraile et al. (2013) [24]. Both *Candida* species showed similar acetic acid production (~0.15 g/L), however they were higher than those obtained with *S. cerevisiae* (0.07 g/L).

Glycerol is another interesting metabolite. The compound has the ability to increase both density and viscosity of the wine [25], and additionally helps to increase the sweet perception of the wine. In our research, no significant differences were found in glycerol production between the control (11.27 g/L) and *C. boidinii* (10.97 g/L) or *C. oleophila* (9.47 g/L). Likewise, no significant differences were observed in the glycerol production between the *Candida* yeasts.

#### 3.2.2. Sequential Fermentation

**Figure 3:**
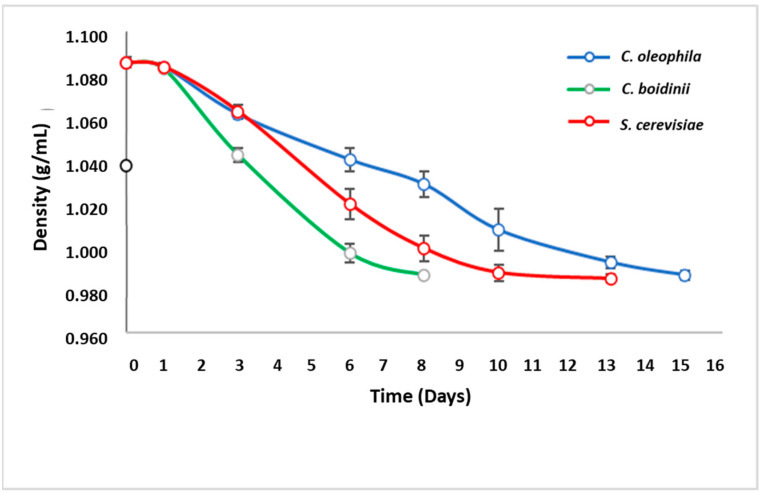
Evolution of substrate consumption through the measurement of density in sequential fermentations (Sauvignon Blanc).

**Figure 4:**
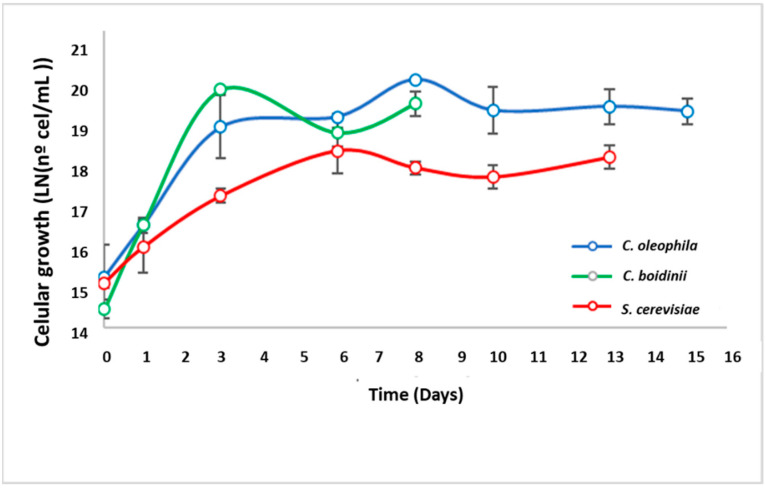
Evolution of cell proliferation in sequential fermentations (Sauvignon Blanc).

Sequential fermentations (NSY inoculation followed by *S. cerevisiae* inoculation) have been reported as positively influencing the wine attributes. Therefore, with the purpose of evaluating the approach with the *Candida* species, sequential fermentations were performed at the laboratory level in Sauvignon Blanc grape juice from the Casablanca Valley (Valparaíso, Chile). The initial density of the grape juice was 1.090 g/mL (22.2 °Brix). The evaluation of the sequential fermentation was evaluated by monitoring of density (Figure 3), cell growth (Figure 4), and the final composition of the grape juice upon fermentation (Table 4 and Table 5).

The fastest fermentation process was observed for the *C. boidinii*–*S. cerevisiae* duo. The inoculation of *S. cerevisiae* occurred when 50% of the available sugars had been consumed, corresponding to day 3 of fermentation (Figure 3), which generated a rapid consumption of sugars upon inoculation of *S. cerevisiae*. This led to a significant shortening of the fermentation process duration to up to 8 days, compared to the monoculture fermentation of *C. boidinii*, which lasted 10 days. In the case of the *C. oleophila-S. cerevisiae* fermentation, the inoculation with *S. cerevisiae* occurred on day 5. This fermentation showed a slower than the control (*S. cerevisiae*) and the *C. boidinii-S. cerevisiae* fermentation, which is consistent with the performance in monoculture fermentations.

Cell growth (Figure 4) showed fluctuations in cell concentration, inherent to these processes, where exponential and stationary phases were also clearly observed. As expected, in the fermentation with *C. oleophila* once *S. cerevisiae* was inoculated, a clear increase in cell concentration was *observed*. However, for *C. bodinii* a drop in the cell concentration from Ln19 to Ln18 CFU/mL was observed, after inoculation with *S. cerevisiae*. Yeast interactions in wine fermentations are usually complex, generating in some cases antagonistic effects that can generate drops in cell concentrations [18,24].

Table 4 and Table 5 show the metabolites production. Ethanol content in sequential fermentations, showed a reduction of 1.5% *v*/*v* for the *C. oleophila*–*S. cerevisiae* fermentation (11.37% *v*/*v*) compared to the control samples (12.87% *v*/*v*). This result was consistent with a prior study, where the ethanol production in *C. oleophila*–*S. cerevisiae* sequential fermentations (12.5% *v*/*v*) yielded 1.0% *v*/*v* less ethanol than the produced by *S. cerevisiae* (13.5%*v*/*v*) [9]. On the other hand, a drop of 0.73% *v*/*v* in ethanol content was observed in the *C. oleophila*–*S. cerevisiae* sequential fermentation (11.37% *v*/*v*) compared to the monoculture fermentation with *C. oleophila* (12.10% *v*/*v*).

For the *C. boidinii*–*S. cerevisiae* fermentation (12.83% *v*/*v*), there was only a reduction of 0.04% *v*/*v* in ethanol content compared to the control sample. Similar results we reported by Aplin et al. (2019) using other NSY followed by a sequential inoculation with *S. cerevisiae*. The authors reported a reduction of about 0.09% *v*/*v* in the ethanol content [10]. On the other hand, the *C. boidinii*–*S. cerevisiae* sequential fermentation (12.83% *v*/*v*) resulted in 0.5% *v*/*v* more ethanol than the monoculture fermentation with *C. boidinii* (12.37% *v*/*v*).

In terms of residual sugar, all sequential fermentations finished with less than 2.5 g/L, showing no significant differences between the fermentations. These results indicate an effective consumption of sugar towards the end of fermentation. This is mainly because the remaining sugar from fermentation only with NSY was consumed at an accelerated rate by the *S. cerevisiae* [21,25].

Table 5 shows the concentration observed for the production of the different fermentation metabolites. In terms of acetic acid, the *C. oleophila*–*S. cerevisiae* and *C. boidinii*–*S. cerevisiae* sequential fermentations showed very low concentrations (less than 0.1 g/L). In the case of the *C. oleophila*–*S. cerevisiae* fermentation, this result differs from the findings of a previous study, where the production of acetic acid was around 0.53 g/L, although in this case the fermentation was done in Carmenere grape juice [9]. On the other hand, the monoculture fermentations produced more acetic acid than the sequential fermentations, for *C. oleophila* 0.15 g/L (monoculture) and 0.03 g/L (sequential), *C. boidinii* 0.14 g/L (monoculture) and not detectable (sequential). It has been reported that some NSY, such as *L. thermotolerans*, can modulate the acidity of wine, through a low production of acetic acid, but an increase in the production of lactic acid [8].

Finally, significant differences were observed in the production of glycerol within the sequential fermentations and also the control (*S. cerevisiae*). The glycerol concentration in the *C. oleophila*–*S. cerevisiae* fermentation (5.17 g/L) was significantly lower than the control *S. cerevisiae* (11.25 g/L). Differences were also observed in comparison with the *C. boidinii*–*S. cerevisiae* (6.77 g/L) fermentation, although the latter showed no significant differences with the *C. oleophila*–*S. cerevisiae* fermentation. Moreover, the glycerol content in the control fermentations was significantly higher than any of the sequential fermentations analyzed. Our results are inconsistent results previously reported, who found that sequential fermentations produce more glycerol than the control with *S. cerevisiae* [11,13,26,27]. In addition, it must be noted that the glycerol produced sequentially (Table 4) was much lower than that produced in monoculture (Table 2). Rolle et al. (2018) [28] and Englezos et al. (2018) [29] indicated that in sequential fermentations, the production of glycerol was higher than in monoculture fermentations, although Englezos et al. worked with *Starmerella bacillaris*. However, the production of glycerol depends on several related factors such as yeast genera and species, as well as environmental factors such as temperature, pH, and nutrients [18,28]. In addition to the above, some *Candida* yeasts such as *C. famata* can produce exopolysaccharides from glucose and/or fructose in the must. These exopolysaccharides have a positive effect on mouthfeel and astringency. These types of compounds can compete with the sensory effects of glycerol [14].

As it expected there are differences between monoculture and sequential fermentations at the laboratory level, both in ethanol production and secondary biochemical compounds. In general terms, it was established that for the case of monoculture fermentation with *C. oleophila*, more ethanol was produced (12.10% *v*/*v*) than in the *C. oleophila*–*S. cerevisiae* sequential fermentation (11.37% *v*/*v*), generating a difference of 0.73% *v*/*v*. On the other hand, the *C. oleophila*–*S. cerevisiae* sequential fermentation showed a difference of 1.5% *v*/*v* of ethanol content in comparison to the control (*S. cerevisiae*), while the monoculture fermentation for *C. oleophila*, only varied by 0.77% *v*/*v* with the control. This was not observed for the monoculture and sequential fermentations with *C. boidinii*, where ethanol production was relatively similar. This suggests that sequential fermentations are a feasible process to obtain reduced ethanol concentration. However, this need to be validated at higher fermentation volumes.

Regarding the production of acetic acid, clear differences between monoculture and sequential fermentations can be observed. While in monoculture fermentation the Candida yeasts produced concentration of acetic acid around 0.14–0.15 g/L, in sequential culture it was reduced to levels of 0.03 g/L for *C. oleophila* and was even lower for *C. boidinii*. Both values lower than the control (0.07 g/L). Similar results were found in other studies where the interaction between NSY and S. cerevisiae produce less acetic acid than pure cultures with NSY [30]. Matraxia et al. (2021) reported that the interaction in co-culture between *H. uvarum* and *S. cerevisiae* resulted in less acetic acid (0.03 g/L) than a pure culture of *H. uvarum* (0.27 g/L) [31]. Conversely, Sadoudi et al. (2012) reported 0.86 g/L of acetic acid in monoculture with C. zemplinina, while the same yeast co-inoculated with S. cerevisiae resulted in 0.51 g/L. In the same study, monoculture of *T. del brueckii* produced 0.65 g/L of acetic acid, in contrast to the interaction of *T. del brueckii* with *S. cerevisiae* with only 0.24 g/L of acetic acid. The presence and concentration of acetic acid is relevant due to its relation with volatile acidity. This parameter can be affected by the maturity (initial sugar concentration) and also the NSY–*S. cerevisiae* interactions, as reported by Dos Santos et al. (2003) [32].

### 3.3. Microvinifications

In order to reproduce a similar fermentation process as the one carried out in wineries, the *Candida* yeasts were used to perform fermentations at the microvinification scale (10 L). Microvinifications were also performed with Sauvignon Blanc grape juice, however the grape juices were acquired from two different locations: Casablanca Valley (Valparaíso, Chile), harvested on April 18, 2020, (23.4 °Brix, density 1.097 g/mL, pH 3.0, titratable acidity 8.64 g/L tartaric acid), and the Curicó Valley (Maule, Chile), harvested on March 10, 2020 (21,7 °Brix, density 1.087 g/mL, pH 3.19, titratable acidity 5.7 g/L tartaric acid). The varieties were selected because they are grown at different geographical locations and harvested at different times. Both monoculture and sequential fermentations were done.

#### 3.3.1. Microvinification with Sauvignon Blanc Grape Juice form the Casablanca Valley

Production of ethanol in microvinifications (monoculture and sequential) with Sauvignon Blanc grape juice from Casablanca Valley did not vary significantly between the fermentation’s systems (Table 6). Ethanol production in monoculture microvinifications varied between 13.57% *v*/*v* (*C. boidinii*) and 13.90% *v*/*v* (*C. oleophila*). The concentration achieved with *C. boidinii* was 0.17% *v*/*v* less than the control, and in the case of *C. oleophila* 0.16% *v*/*v* higher than the control (13.74% *v*/*v*). The ethanol content in the sequential microvinifications, varied between 13.20% *v*/*v* (*C. boidinii–S. cerevisiae*) and 13.12% *v*/*v* (*C. oleophila–S. cerevisiae*), that is 0.54 and 0.72% *v*/*v* of ethanol content lower than the control, respectively. These results were unexpected as, given the trends seen in the prior experiments, a significantly reduction in ethanol production was expected. While statistically significant reductions were not seen, an average lower ethanol production was observed for fermentations in the presence of *Candida* yeasts.

This might indicate that both the complexity of the grape juice, and to a greater extent the scaling of the fermentation process along with winery practices, have significant influence on the concentration of metabolic compounds. In a study performed by Liccioli et al. (2011) [33], they determined that with lower fermentation volumes (laboratory scale) there is a greater rate of volatilization of ethanol. On the other hand, at higher volumes, such as that of a microvinification or at the winery level, the volatilization rates are much lower. This could explain the more reduced ethanol concentrations in laboratory-scale fermentations versus the microvinifications in our study. As expected, the sequential microvinifications produced less ethanol than monoculture ones. Thus, for example, the production of ethanol was reduced by 0.37% *v*/*v* from monoculture to sequential (13.57 to 13.20% *v*/*v*) for the *C. boidinii* yeast, and by 0.78% *v*/*v* for *C. oleophila* (13.90 to 13.12% *v*/*v*).

Regarding to residual sugars, as expected, glucose was consumed almost entirely, achieving remaining values ranging from 0.2 to 0.4 g/L. On the other hand, the residual sugar was mostly constituted by fructose, with levels lower than 1.75 g/L. This phenomenon is common in fermentations with pure *S. cerevisiae*, or in sequential fermentations with *S. cerevisiae*. On the other hand, the fructophilic nature of the *C. boidinii* and *C. oleophila* was observed, whose participation in monoculture and sequential fermentations resulted in significantly lower residual sugar than in the control, similarly to what has been reported previously [10,30].

#### 3.3.2. Microvinification with Sauvignon Blanc Grape Juice form the Curicó Valley

Table 7 summarizes the results observed for these microvinifications. In terms of ethanol production, while no significant differences were observed, it is interesting to see that all fermentations in which the *Candida* yeasts produced less ethanol. The control (*S. cerevisiae*) resulted in 11.95% *v*/*v* of ethanol, while the microvinifications with *C. oleophila* resulted in 11.41% *v*/*v*, that is, a difference of 0.54% *v*/*v*. In the case of *C. boidinii* (11.51% *v*/*v*), the difference was lower achieving a value of 0.44% *v*/*v*.

As for sugars consumption, glucose levels reached not significantly different final values, ranging from 0.06 to 0.11. On the other hand, the remaining fructose was significantly higher, in particular for the *C. oleophila*–*S. cerevisiae* duo, that ended with 2.81 g/L.

Regarding the production of glycerol, the sequential fermentation with *C. boidinii* was significantly higher than the rest with 8.51 g/L, while the sequential fermentation with *C. oleophila* generated 5.37 g/L. The control (*S. cerevisiae*) produced a quantity of glycerol of 6.7 g/L, significantly higher than *C. oleophila*, but significantly lower than *C. boidinii*. Differences in glycerol concentrations might be attributed to differences in the activation of the protective mechanisms of each fermentation duo. The increase in glycerol production is commonly due to the fact that several genera of yeast use it as a protective metabolite in the face of adverse ethanol concentrations [30].

In terms of acetic acid production, the lower production was observed for *C. oleophila* –*S. cerevisiae* duo (0.11 g/L), while the *C. boidinii*–*S. cerevisiae* duo resulted in 0.46 g/L. However, in all cases, the level of acetic acid was lower than the sensory threshold reported for the acid of approximately 0.7 g/L [34].

Summarizing, on average, the ethanol production with *Candida* yeasts in microvinifications was higher than that obtained at the laboratory scale, which would indicate that the scaling of the fermentation process significantly influences the ethanol content of the fermentations. Meanwhile, the sequential fermentations with either of the two *Candida* yeasts resulted in less ethanol than the monoculture fermentations; for *C. boidinii* from 13.57% *v*/*v* (monoculture) to 13.20% *v*/*v* (sequential), that is, a difference in ethanol content of 0.37% *v*/*v*. In the case of *C. oleophila*, from 13.90% *v*/*v* (monoculture) to 13.12% *v*/*v* (sequential), that is, a difference in ethanol content of 0.78% *v*/*v*.

Evidently, sugar concentration (grape maturity level) influences the final wine characteristics. Initial sugar concentration between the Casablanca and Curicó grape juices were 23.4 and 21.7 °Brix, respectively. In terms of ethanol production, the sequential fermentation from Casablanca produced average concentrations of 13.82% *v*/*v* (*S. cerevisiae*: 13.74% *v*/*v*; *C. boidinii*: 13.20% *v*/*v*; *C. oleophila* 13.12% *v*/*v*). On the other hand, fermentations under the same conditions from the Curicó grape juice produced average concentrations of 11.27% *v*/*v* (*S. cerevisiae*: 11.95% *v*/*v*; *C. boidinii*: 11.51% *v*/*v; C. oleophila* 11.41% *v*/*v*). This would indicate to higher sugar concentration in grape juices, the *Candida* yeasts are more efficient to reduce ethanol.

## 4. Conclusions

To our knowledge, this is the first report of the use of *Candida* yeast at larger volumes (10 L) than laboratory scale, following a fermentation process that mimics the practices used at the winery scale, which is relevant for their potential use at industrial scale. Furthermore, a lyophilized starter culture was used opening a potential industrialization of these native yeasts.

In summary, and comparing the different medium, scales and fermentation strategies, it can be concluded the following: (1) The *Candida* yeasts studied showed a lower ethanol yield in the G/F medium; (2) the management and control of fermentation conditions influences the final characteristics of the wine; (3) *Candida* yeasts products wines with less ethanol content when grapes juices with higher sugar concentration are used, and a sequential fermentation is followed; (4) the results of microvinification are more representative of industrial winemaking.

The results presented in this study are the first reports for the use of non-*Saccharomyces* yeasts belonging to the *Candida* genera at a scale and procedure that mimics the ones used at the winery setting. However, more studies are needed, such as the study of the volatile profile, and other grape varieties.

## Figures and Tables

**Table 1:** Fermentations results using a synthetic glucose–fructose medium.

Yeast	Ethanol (% *v*/*v*)	Sugar Consumption (%)	Yield (g Ethanol/g Glucose)
*C. oleophila*	4.20 ± 0.53 ^a^	71.77 ± 2.41 ^a^	0.25 ± 0.02 ^a^
*C. boidinii*	6.40 ± 0.20 ^b^	99.89 ± 1.31 ^b^	0.24 ± 0.04 ^a^
*S. cerevisiae*	13.90 ± 0.65 ^c^	99.92 ± 1.11 ^b^	0.45 ± 0.04 ^b^

Data reported are mean values ± standard deviations. Mean values with different letters in the same column are significantly different (*p* < 0.05).

**Table 2:** Monoculture fermentations of Sauvignon Blanc from Casablanca Valley (Valparaíso, Chile).

Yeast	Ethanol (%*v*/*v*)	Residual Sugar (g/L)	Yield (g Ethanol/g Glucose)	G/F	Days
*C. oleophila*	12.10 ± 0.10 ^a^	2.70 ± 0.26 ^a^	0.347	1.074	15
*C. boidinii*	12.37 ± 0.06 ^b^	2.17 ± 0.84 ^a^	0.354	1.070	10
*S. cerevisiae*	12.87 ± 0.16 ^c^	2.50 ± 0.17 ^a^	0.368	1.072	13

Data reported are mean values ± standard deviations. Mean values with different letters in the same column are significantly different (*p* < 0.05).

**Table 3:** Secondary compounds of monoculture fermentations of Sauvignon Blanc from Casablanca Valley (Valparaíso, Chile).

Yeast	g/L	
	Acetic Acid	Glycerol
*C. oleophila*	0.15 ± 0.15 ^a^	9.47 ± 1.17 ^a^
*C. boidinii*	0.14 ± 0.19 ^a^	10.97 ± 0.84 ^a^
*S. cerevisiae*	0.07 ± 0.02 ^a^	11.27 ± 0.46 ^a^

Data reported are mean values ± standard deviations. Mean values with different letters in the same column are significantly different (*p* < 0.05).

**Table 4:** Sequential fermentations of Sauvignon Blanc from Casablanca Valley (Valparaíso, Chile).

Yeast	Ethanol (%*v*/*v*)	Residual Sugar (g/L)	Yield (g Ethanol/g Glucose)	G/F	Days
*C. oleophila*	11.37 ± 1.46 ^a^	2.03 ± 0.25 ^a^	0.368	1.072	15
*C. boidinii*	12.83 ± 0.55 ^a^	2.40 ± 0.20 ^a^	0.368	1.071	8
*S. cerevisiae*	12.87 ± 0.12 ^a^	2.25 ± 0.17 ^a^	0.358	1.072	13

Data reported are mean values ± standard deviations. Mean values with different letters in the same column are significantly different (*p* < 0.05).

**Table 5:** Secondary compounds of sequential fermentations of Sauvignon Blanc grape juice from Casablanca Valley (Valparaíso, Chile).

Yeast	g/L	
	Acetic Acid	Glycerol
*C. oleophila*	0.03 ± 0.03 ^a^	5.17 ± 0.76 ^a^
*C. boidinii*	NDL	6.77 ± 0.45 ^a^
*S. cerevisiae*	0.07 ± 0.02 ^a^	11.25 ± 0.46 ^b^

Data reported are mean values ± standard deviations. Mean values with different letters in the same column are significantly different (*p* < 0.05).

**Table 6:** Composition of Sauvignon Blanc wine obtained by microvinification with grapes from the Casablanca Valley (Valparaíso, Chile).

Yeast	Fermentation	Ethanol % *v*/*v*	g/L					
			Fructose	Glucose	Glycerol	Acetic Acid	Malic Acid	Lactic Acid
*S. cerevisiae*	Control	13.74 ± 0.18 ^a^	1.73 ± 0.20 ^a^	0.39 ± 0.11 ^a^	7.26 ± 0.27 ^a^	0.29 ± 0.12 ^a^	2.42 ± 0.28 ^a^	0.09 ± 0.03 ^a^
*C. boidinii*	Monoculture	13.57 ± 0.66 ^a^	0.82 ± 0.00 ^ab^	0.23 ± 0.04 ^b^	6.14 ± 0.10 ^c^	0.23 ± 0.03 ^ab^	2.22 ± 0.03 ^ab^	ND
	Sequential	13.20 ± 0.84 ^a^	1.32 ± 0.40 ^ab^	0.22 ± 0.02 ^c^	6.74 ± 0.11 ^b^	0.21 ± 0.05 ^ab^	2.23 ± 0.02 ^ab^	0.10 ± 0.01 ^a^
*C. oleophila*	Monoculture	13.90 ± 0.98 ^a^	1.07 ± 0.26 ^ab^	0.21 ± 0.06 ^b^	6.55 ± 0.19 ^b^	0.29 ± 0.06 ^a^	2.33 ± 0.14 ^ab^	ND
	Sequential	13.12 ± 1.15 ^a^	0.60 ± 0.15 ^b^	0.099 ± 0.02 ^b^	6.71 ± 0.16 ^b^	0.16 ± 0.02 ^b^	2.37 ± 0.15 ^ab^	0.17 ± 0.01 ^a^

Data reported are mean values ± standard deviations. Mean values with different letters in the same column are significantly different (*p* < 0.05). The glycerol concentrations observed in both monoculture and sequential fermentations were lower than the values observed for the control.

**Table 7:** Composition of Sauvignon Blanc wine obtained by sequential microvinification with grapes from the Curicó (Maule, Chile).

Yeast	Ethanol % *v*/*v*	g/L					
		Fructose	Glucose	Glycerol	Acetic Acid	Malic Acid	Lactic Acid
*S. cerevisiae*	11.95 ± 0.14 ^a^	1.79 ± 0.40 ^a^	0.06 ± 0.03 ^a^	6.70 ± 0.87 ^a^	NDL	2.00 ± 0.22 ^a^	0.18 ± 0.03 ^ab^
*C. boidinii*	11.51 ± 0.84 ^a^	2.04 ± 0.29 ^a^	0.08 ± 0.02 ^a^	8.51 ± 0.12 ^c^	0.46 ± 0.29 ^b^	1.75 ± 0.01 ^b^	0.05 ± 0.02 ^a^
*C. oleophila*	11.41 ± 0.55 ^a^	2.81 ± 0.83 ^a^	0.11 ± 0.02 ^a^	5.37 ± 0.19 ^b^	0.11 ± 0.01 ^a^	0.11 ± 0.08 ^c^	0.21 ± 0.14 ^b^

Data reported are mean values ± standard deviations. Mean values with different letters in the same column are significantly different (*p* < 0.05). NDL: No Detectable Level.

## Data Availability

Data can be obtained freely by contacting the corresponding author.
